# Review of Abnormal Self-Knowledge in Major Depressive Disorder

**DOI:** 10.3389/fpsyt.2019.00130

**Published:** 2019-03-28

**Authors:** Yixue Lou, Yi Lei, Ying Mei, Paavo H. T. Leppänen, Hong Li

**Affiliations:** ^1^College of Psychology and Sociology, Shenzhen University, Shenzhen, China; ^2^Faculty of Education and Psychology, University of Jyväskylä, Jyväskylä, Finland; ^3^Center for Neurogenetics, Shenzhen Institute of Neuroscience, Shenzhen, China

**Keywords:** major depressive disorder, self-knowledge, abnormality, behavioral abnormality, neurological abnormality

## Abstract

**Background:** Major depressive disorder (MDD) is an affective disorder that is harmful to both physical and mental health. Abnormal self-knowledge, which refers to abnormal judgments about oneself, is a core symptom of depression. However, little research has summarized how and why patients with MDD differ from healthy individuals in terms of self-knowledge.

**Objective:** To gain a better understanding of MDD, we reviewed previous studies that focused on the behavioral and neurological changes of self-knowledge in this illness.

**Main Findings:** On the behavioral level, depressed individuals exhibited negative self-knowledge in an explicit way, while more heterogeneous patterns were reported in implicit results. On the neurological level, depressed individuals, as compared with non-depressed controls, showed abnormal self-referential processing in both early perception and higher cognitive processing phases during the Self-Referential Encoding Task. Furthermore, fMRI studies have reported aberrant activity in the medial prefrontal cortex area for negative self-related items in depression. These results revealed several behavioral features and brain mechanisms underlying abnormal self-knowledge in depression.

**Future Studies:** The neural mechanism of implicit self-knowledge in MDD remains unclear. Future research should examine the importance of others' attitudes on the self-concept of individuals with MDD, and whether abnormal self-views may be modified through cognitive or pharmacological approaches. In addition, differences in abnormal self-knowledge due to genetic variation between depressed and non-depressed populations remain unconfirmed. Importantly, it remains unknown whether abnormal self-knowledge could be used as a specific marker to distinguish healthy individuals from those with MDD.

**Conclusion:** This review extends our understanding of the relationship between self-knowledge and depression by indicating several abnormalities among individuals with MDD and those who are at risk for this illness.

## Introduction

Major depressive disorder (MDD) is a complicated affective disease characterized by abnormal clinical symptoms, including neurovegetative dysfunction (appetite or sleep disturbances), cognitive dissonance (inappropriate guilt, feelings of worthlessness), aberrant psychomotor activities (agitation or retardation) (1), and elevated suicide risk (2, 3). According to the World Health Organization, there are approximately 350 million people suffering from depression worldwide (4). In a recent survey, the proportion of years lived with disability (YLDs) caused by MDD was 4.2%, approximately 34.1 million of the total YLDs (5). Thus, MDD is thought to be a major global cause of disease burden and human suffering (5–7).

Abnormal perception and understanding of the self is a core symptom of MDD (1). This includes abnormal processes and/or representations involved in being aware of the self, abnormal knowledge about the self, and/or abnormal judgments about the self (National Institute of Mental Health; NIMH). As a sub-construct of perception and understanding of the self, self-knowledge, which refers to the ability to make judgments about one's current cognitive or emotional internal states, traits, and/or abilities (NIMH), is also impaired in individuals with MDD (8–11). For instance, individuals with MDD, unlike non-depressed healthy individuals, often exhibit negative self-evaluation, inappropriate self-blame, and excessive self-criticism (8, 12).

**Figure 1:**
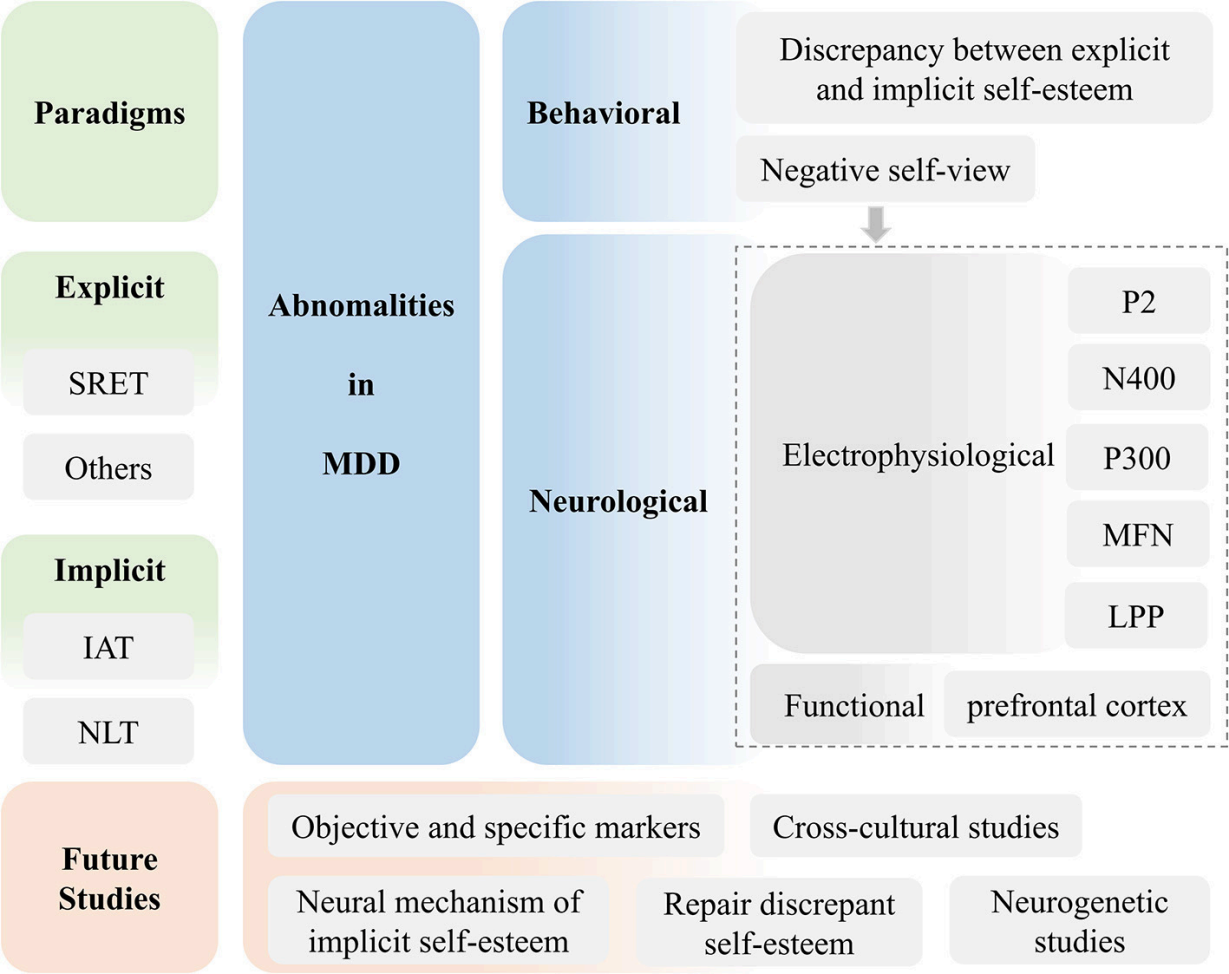
Framework of the current review.

Although researchers have increasingly begun exploring abnormal self-knowledge in depression, few have compared existing findings in a single study. To enable a better understanding of how and why patients with MDD differ from healthy individuals in terms of self-knowledge, the current review focused on previous studies that examined behavioral patterns and brain mechanisms underlying abnormal self-knowledge in depression. Both explicit and implicit self-knowledge, which reflect conscious and unconscious self-views respectively, were discussed. Various abnormalities such as abnormal brain responses and aberrant neural circuits were illustrated. Furthermore, the present review pointed out some possible directions for future clinical studies (see Figure 1).

## Literature

### Literature Review

A search of previous studies published between January 1960 and August 2018 was conducted using the databases Web of Science and PubMed. Self-knowledge is defined as a construct that includes self-evaluation, self-esteem, and self-reference. Thus, the search terms were designed as follows: “depression AND self-evaluation,” OR “depression AND self-esteem,” OR “depression AND self-reference.” Search filters were set for publications written in English. Empirical research and reviews that examined the role of self-evaluation, self-attitude, self-view, self-reference, and/or self-esteem in MDD were found.

### Eligibility Criteria

We screened for inclusion based on titles and abstracts, and again using full text. To be included, previous studies had to focus on behavioral and neurological changes of self-knowledge in MDD. All publications had to be reported on clinical populations currently or previously diagnosed with MDD, or populations who were currently in a depressive episode, regardless of gender and age. Conference abstracts were excluded if they were not published in a scientific journal. Publications were also excluded if they were published in a language other than English (see Supplementary Figure 1).

## Paradigms

The majority of the research conformed to one of two methods. Specifically, these were explicit and implicit research paradigms.

### Explicit Paradigms

Explicit methods are used to assess individuals' self-attitudes by using self-reported measures such as direct self-evaluation. The most commonly used explicit methods are the Self-Referential Encoding Task (SRET) (13) and self-reported questionnaires (14, 15).

#### Self-Referential Encoding Task, SRET

**Figure 2:**
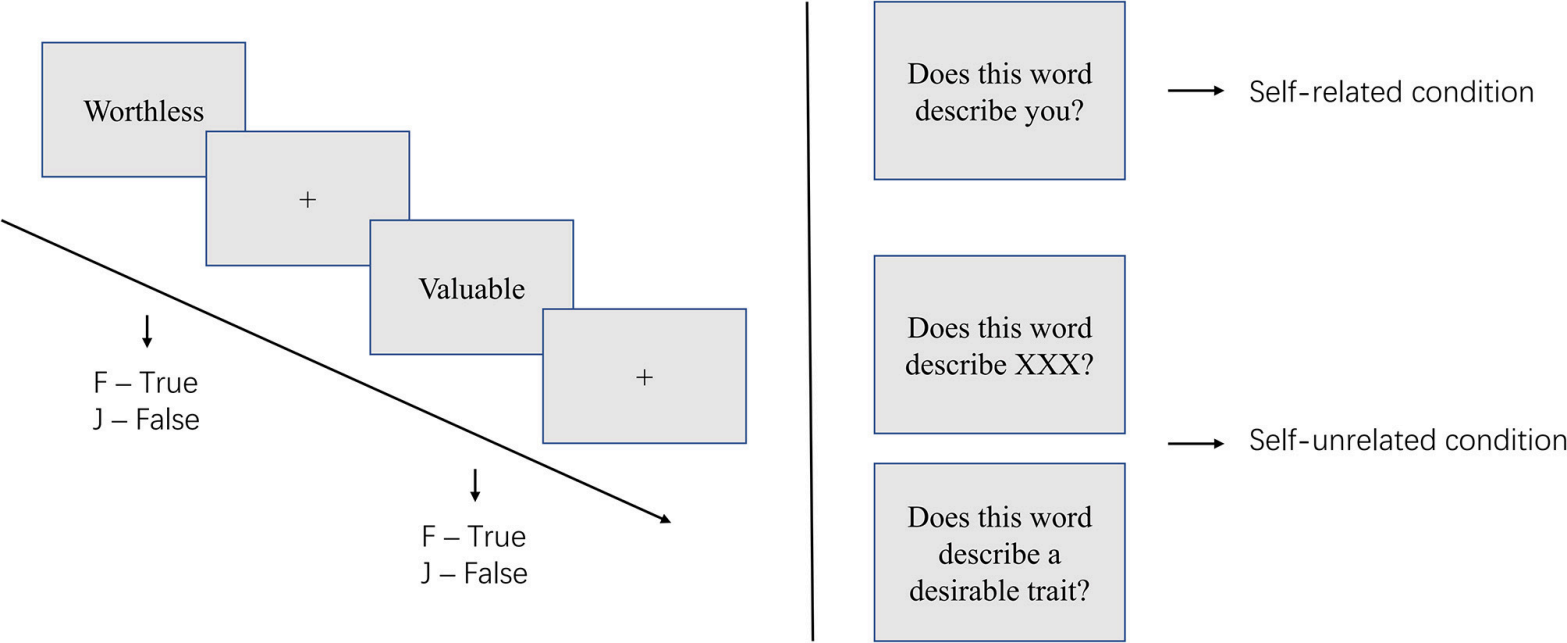
Illustration of the self-referential encoding task (SRET).

The self-referential encoding task (SRET) was designed to examine one's self-attitude (13, 16). Theoretically, individuals are more sensitive to information that is encoded as strongly related to oneself (17). Thus, self-related stimuli commonly display better recall and recognition performance, when compared to other-related stimuli (18). In the SRET, researchers present participants with positive and negative personality trait words, and ask them to decide whether each trait describes themselves (self-related condition), a familiar other (other-related condition) (19–22), or a socially desirable trait (semantic encoding condition; see Figure 2) (10, 23). After the judgment, the participants were asked to recall or recognize all the trait words that had been presented to them.

Individuals with positive self-attitudes, such as feelings of self-value, commonly endorse more positive traits relative to negative traits as self-describing, and show better recall and recognition rates of these words (18). Conversely, negative self-attitudes, such as feelings of worthlessness in individuals with MDD, often lead to more endorsement of negative traits and, in turn, better memory performance of these words (18, 24).

#### Other Explicit Approaches

Direct self-report questionnaires are often used in studies of depressive self-knowledge. For instance, researchers have used the Rosenberg Self-Esteem Scale (RSES) to measure explicit self-esteem in depression (15). In addition, the self-worth subscale of the World Assumption Scale (25) and the self-acceptance subscale of the Scales of Psychological Well-Being (26) are used to assess explicit self-attitude in depression. Moreover, the Beck Depression Inventory (BDI), which is commonly used to measure the depressive state, also contains self-evaluation factors, such as the self-blame factor, in its items (14).

### Implicit Paradigms

The efficacy of explicit methods is debated by some researchers for the following reasons. First, according to cognitive theory, the self-concept involves automatic processes that occur without reflection and/or logical reasoning accessible within the conscious mind (27). Second, direct self-appraisal might be affected by social desirability and cultural differences (28, 29). In brief, explicit methods may not accurately reflect a person's real attitude about him/herself (30, 31). Thus, implicit paradigms were introduced into self-knowledge studies (32–34). The most commonly used implicit paradigms are the Implicit Association Test (IAT) (35, 36) and the Name-Letter Test (NLT) (37, 38).

#### Implicit Association Task, IAT

The self-evaluation IAT (sIAT) is a paradigm that has been commonly used to examine implicit self-attitudes of depression (39, 40). In the sIAT, it is assumed that information that is compatible with one's implicit attitude would be better processed as compared to that which is incompatible (36). Thus, participants are asked to complete two types of categorization (compatible and incompatible) by using a two key-press system. In the compatible condition, self-related stimulus words (e.g., one's own name or date of birth) shared the same key with “valuable” personality trait words (e.g., competent), while self-unrelated words (e.g., other's name or non-meaningful date) shared another key with “worthless” personality trait words (e.g., unsuccessful). The incompatible condition was reverse coded (34, 41, 42) (Table 1).

**Table 1:** Illustration of the self-evaluation Implicit Association Task (sIAT).

**Task**	**Categorization**	**Stimulus**	**Key-press**
Compatible	Self-related/unrelated words	Self	F
		Other	J
	Personality trait words	Valuable	F
		Worthless	J
Incompatible	Self-related/unrelated words	Self	F
		Other	J
	Personality trait words	Valuable	J
		Worthless	F

Differences in reaction times (RTs) and accuracy (ACC) between compatible and incompatible conditions were analyzed. Typically, the condition that is congruent with one's implicit self-attitude should show better performance when compared to the incongruent one. For instance, individuals with positive self-bias should demonstrate a faster and more accurate response in the compatible condition, relative to the incompatible condition (43, 44), while the negative self-attitude found in depression should lead to an opposite pattern.

#### Name-Letter Test, NLT

The name-letter test (NLT) has also been used in previous studies to measure implicit self-attitudes (38, 45–47). In the NLT, researchers presented participants with the 26 letters of the alphabet one-by-one, and asked them to judge the attractiveness or likability of each letter, relying on their first, intuitive reaction (48). According to the name letter effect, one's initial is thought to be highly associated with the self (49, 50). Thus, under the influence of positive self-bias, non-depressed individuals should show a rational preference toward their initials relative to other letters, even though they are generally unaware of this effect (38). However, an opposite pattern may be true for individuals with MDD (33).

The name letter effect has been shown to be a cross-cultural phenomenon, since it has also been reported in Thai, Japanese, and Korean studies (51–53). Thus, the NLT qualifies as an indirect assessment of self-attitude in depression (33).

## Main findings

By using the aforementioned paradigms, researchers have found abnormal behavioral patterns and brain responses in individuals with MDD, when compared to non-depressed, healthy controls. Evaluation of the quality of included studies was listed in Supplementary Table 1.

### Behavioral Abnormalities

Behavioral abnormalities include explicit/conscious and implicit/unconscious behaviors that have been observed in depression.

#### Explicit: Negative Self-View

At the explicit level, previous behavioral research has revealed a negative self-view in depression, as compared with a non-depressed healthy population. For instance, healthy individuals typically exhibit positive attitudes about themselves (54–57). For instance, they often attribute themselves with more positive, rather than negative, personality traits (54, 58), so that their self-esteem may be protected (18, 59). However, individuals with depression typically demonstrate an abnormally negative self-view (1, 60, 61).

For instance, under the influence of negative self-knowledge, individuals with MDD show less positive self-bias, less self-confidence, and lower self-esteem (62–65), as well as excessive self-criticism, negative self-evaluation, inappropriate self-blame, and shame (8, 12, 66–68). This negative self-representation has been associated with greater self-reported depression (69, 70), poor and slower recovery from a major depressive episode (71, 72), and higher probability of suicide attempt (73, 74). In addition, individuals with higher self-esteem may exhibit sudden improvements in depressive symptomatology even without treatment (75), while lower self-esteem is thought to be a prospective risk factor for depressive symptoms from young adulthood to old age (76–78).

In the SRET, individuals with depression, relative to healthy controls, endorsed more negative trait words as self-described, and showed faster response, better recall performance, and increased recognition rate for these words (9, 23, 79, 80). In a longitudinal study Derry and Kuiper (13), found that such negative self-bias might be a specific symptom in currently depressed patients, since the recall rate of negative self-related words decreased after recovery from the illness.

#### Implicit: Discrepancy Between Explicit and Implicit Self-Esteem

Although a large number of studies have indicated a lower self-attitude in MDD, relative to healthy individuals, at an explicit level (8–10, 20), more heterogeneous patterns have been reported in implicit studies (34, 36, 41, 42, 81).

For instance, when using the IAT and/or NLT to measure implicit self-esteem (ISE) and RSES to assess explicit self-esteem (ESE), some researchers have observed both low ESE and ISE in currently depressed individuals (39, 40, 42) and chronically depressed individuals with early onset (33), relative to never depressed healthy controls. However, more researchers have reported a normal ISE combined with a lower ESE in individuals with current depression (41, 42, 82–85), previous depression (41), remitted depression (11, 39, 86), and chronic depression with late onset (33), when compared to non-depressed individuals. Some researchers have even observed higher ISE and lower ESE in current depression (83, 85, 87) and previous depression (34, 82).

The discrepancy between explicit and implicit self-esteem, especially the combination of low ESE and high ISE, is thought to be associated with internalizing problems such as affective disorders (88–92). For major depression, such a discrepancy seems to be more severe in depressed individuals with suicidal ideation relative to those without such ideation (42). Moreover, depressed patients with congruent self-esteem, compared to those with incongruent self-esteem, exhibited better recovery from the illness throughout antidepressant treatment (93).

### Neurological Abnormalities

Neurological abnormalities include abnormal electrophysiological responses and aberrant functional neural activities. These abnormalities were all detected using the SRET.

#### Abnormal Electrophysiological Response

To explore the brain mechanism of negative self-knowledge in depression, electroencephalography (EEG) technology was used in conjunction with the SRET. By collecting the event-related potentials (ERPs) during the SRET, researchers attempted to identify the key ERP components that are involved in negative self-referent processing in MDD.

For instance, Shestyuk and Deldin (62) observed increased P2 component, which was quantified as a positive peak in the 200- to 300-ms time window poststimulus, in individuals with depression while processing negative, relative to positive, self-referential items. The opposite, however, was true for the non-depressed healthy controls. A recent study reported decreased N400 amplitude, which was measured as mean voltage of the ERP average between 350–500 ms, in individuals with depression, as compared with healthy controls, in negative self-referent processing (9). Regarding the latter component, Poulsen et al. (94) found an attenuated or absent MFN response between 260 ms and 480 ms in depression, relative to non-depressed controls, when specifically endorsing negative trait descriptors. However, in a recent study, depressed individuals were found to exhibit enhanced MFN for both positive and negative endorsement (95). Consistently, an attenuated P300 response from 300- to 600- ms was observed in both of these two studies (94, 95). Concerning the more delayed late positive potential (LPP), larger LPP amplitudes were detected following negative vs. positive endorsement in depressed adults (62, 96), depressed adolescents (8), and young girls who were vulnerable to depression (97), when compared to healthy controls.

In these studies, the P2 component is thought to be related to automatic semantic processes (98). Thus, an increased P2 reflects a stronger automatic attentional capture and orientation in patients with depression under the negative, relative to positive, self-related condition (62). The N400 component was interpreted to be influenced by semantic memories about the self, and could be reduced by greater association of the stimuli with a preceding self-related context (99, 100). Therefore, this result indexed a congruent pattern between negative semantic memories and the self-concept in individuals with depression (9). In addition, the MFN is thought to be associated with early cognitive evaluation during self-referential processing (95). The altered MFN response may reflect abnormal self-evaluation among clinically depressed individuals. The greater P300, which is evoked by a saliency effect of self-referential information and positive affect (101), was attenuated in depression. One possible interpretation is that it was possibly associated with a chronically negative self-view in this population (95). Last, an increased LPP amplitude, which is associated with effortful encoding (102), indicates that individuals with depression engage more cognitive effort in processing self-related negative, relative to positive, items (62).

In all, in the time domain, abnormal self-knowledge in depression could be reflected in early phases of self-related processing, such as automatic attention and orientation toward negative self-descriptive items (62). Retrieval of negative memories about the self could also be involved (9). For later phases of self-referential processing, an attenuated bonding between positive affect and the self may be associated with negative self-view in depression (95). Furthermore, depressed individuals seem to engage more cognitive effort in negative, instead of positive, self-reference (62).

#### Abnormal Functional Neural Activities

The high spatial resolution of functional MRI technology makes it possible for researchers to determine abnormal brain activities in depression during the SRET. Several fMRI studies, thus, have suggested that the prefrontal cortex and its sub-regions might be abnormal in individuals with MDD (103). The prefrontal cortex is thought to play an important role in self-referential processing (104). In particular, dysfunction within the medial prefrontal cortex (mPFC) and in the circuits that connect the mPFC to other cortical and limbic structures is responsible for the cognitive dissonance found in depression (103).

For instance, the cortical midline structures (CMS), such as the mPFC, are critical for self-referential processing in healthy individuals (17, 105), adult patients (106–108), and adolescent patients with MDD (109). However, aberrant activity in the mPFC was reported in depression when compared to healthy controls (17, 23, 106). Additionally, Yoshimura et al. (108) found that individuals with depression, relative to healthy controls, exhibited hyperactivity in the mPFC and the rostral anterior cingulate cortex (rostral ACC) during self-referential processing of negative personality traits; such activity was shown to be associated with depressive symptoms (108).

Furthermore, abnormal activities of other sub-regions of the prefrontal cortex were also observed during the processing of self-related negative stimuli in depression (10, 23). For instance, by using the SRET, researchers found significantly higher activation of the central mPFC and significantly lower activation of the dorsal mPFC in depression, relative to healthy controls, during the self-referential condition (10). Local connectivity of the dorsal mPFC was also reduced during self-reflection in depressed adolescents (109). The activity of the dorsolateral prefrontal cortex (dlPFC) was also involved in self-referential processing in depression, but was absent in healthy controls (23). In addition, a meta-analysis revealed hyperactivation in the ventromedial prefrontal cortex (vmPFC) within major depression during resting state, which was discussed as a neural reflection of self-referential processing (110).

Therefore, aberrant activity of the prefrontal cortex and its sub-regions could index the abnormal brain activity that is a hallmark of depression, specifically during the processing of self-referential stimuli. In particular, hyperactivity in the mPFC during negative self-referential processing could possibly even be associated with the severity of depressive symptoms.

## Discussion

According to previous studies, abnormal self-knowledge, which is commonly found in MDD, is mainly reflected in abnormal behaviors and abnormal neurological responses during self-evaluation, self-esteem, and/or self-referential processing.

At the behavioral level, abnormal self-knowledge could be indexed by a negative explicit self-view (13, 80) and discrepant self-esteem, which involves relatively higher implicit self-esteem and lower explicit self-esteem (11, 33, 34, 111). Furthermore, a greater discrepancy between implicit and explicit self-esteem is related to more severe MDD, or a higher possibility of being affected by the illness (42, 111).

At the neurological level, several abnormalities have been found during abnormal self-referential processing, by using electrophysiological technology (8, 9, 62) and fMRI technology (10, 108, 112, 113). For instance, for abnormal electrophysiological processing, enhanced P2 and LPP and decreased N400 amplitudes were all detected in depression, relative to non-depressed controls, in the SRET. For aberrant brain activities, higher activation of the central mPFC, lower activation of the dorsal mPFC (10), and aberrant activity of the dlPFC (23) during self-referential processing can also distinguish MDD, as well as indicate the severity of symptoms.

By using the indexes above, researchers and clinicians could distinguish patients with MDD and non-depressed individuals more objectively and effectively. However, caution should be exercised for several reasons. First, some of the studies involved limited samples and poor replications. For instance, abnormalities in P2 and LPP amplitude in MDD were reported in a study with 17 patients with current depression, 17 patients with remitted depression, and 18 controls, and abnormalities of N400s were reported in a study including 16 patients with MDD and 16 controls. Considering this issue, larger samples are needed to confirm changes of electrophysiological response during depressive self-referential processing.

Second, abnormal self-knowledge is only one component of MDD, despite being a core feature. Behavioral abnormalities may not be sensitive and specific for MDD, since they are affected by non-clinical factors such as personality traits (114–117). Thus, more evidence is needed to confirm the behavioral abnormalities identified in the current review.

Third, although we reviewed various investigations that focused on abnormal self-knowledge in depression, a classical review is relatively less objective compared with a systematic meta-analysis.

## Future studies

In the exploration of self-knowledge in depression, there are still many unanswered questions. First, although the discrepancy between explicit and implicit self-esteem in depression has been confirmed by several previous studies (11), and the neural mechanism of explicit self-esteem has been richly explored (8, 10, 23, 108), little is known about the neural basis of implicit self-esteem in depression, suggesting the need for further research.

Second, it remains unclear whether the pattern of self-knowledge in patients with depression would be different in a cross-cultural context. For instance, collectivism of eastern Asia, relative to individualism in Western culture, allows individuals to view themselves as dynamic entities that are continually defined by their social context and relationships (118). Thus, in Eastern cultures, judgment by important others about oneself, which is currently ignored in self-related studies, plays a critical role in the quality of one's self-view (119). Indeed, the development of self-knowledge relies not only on one's reflection of the self, but also on how important others evaluate the individual (22, 58, 119–121).

Third, some previous neurogenetic research explored the association between different gene types and abnormal self-knowledge in depression, and found that the serotonin transporter promoter polymorphism (5-HTTLPR) played a crucial role in susceptibility to developing depression (122). In a recent study, Ma et al. (21) reported a modulation effect of the 5-HTTLPR polymorphism in brain activities associated with negative self-knowledge in depression. It was suggested that the s allele of 5-HTTLPR could possibly be a risk factor for individuals vulnerable to depression (21). However, differences in abnormal self-knowledge due to genetic variation between healthy and depressed populations remains unconfirmed, calling for further research.

Fourth, to repair discrepant self-esteem found in depression, which involves low explicit and high implicit self-esteem, the development of cognitive and/or medical approaches is needed to enhance explicit self-attitudes. A previous study indicated that depression can be prevented or reduced by interventions that improve explicit self-esteem (123–126). For example, researchers have utilized positive self-images (127) and mindfulness (128, 129) to realize an improvement of both explicit and implicit self-esteem. It is possible that these methods can also be used to diminish the discrepancy of self-esteem found in depression. Furthermore, since the s allele of 5-HTTLPR may elevate the risk of developing depression (21), it is reasonable to consider whether the use of selective serotonin reuptake inhibitors (SSRIs) could enhance self-satisfaction (130–132).

Finally, to conquer complex diseases such as MDD, the National Institute of Mental Health (NIMH) has raised the importance of identifying clinically useful biomarkers and behavioral indicators that predict change across the trajectory of illnesses (19). However, the most fundamental challenge is to identify these diseases effectively. In the diagnosis of MDD, the most commonly used measurements are structured interviews and/or depression inventories (133), which are relatively subjective and require researchers to be professionally trained. To facilitate the identification of objective criteria for MDD diagnosis, it must be determined whether abnormal self-knowledge can be used as an objective and specific marker for identifying MDD. For this purpose, patterns of abnormal self-knowledge should be compared between MDD and other mental disorders, such as bipolar disorder.

## Conclusion

MDD is a main cause of disease burden worldwide (6, 7), and abnormal self-knowledge is one of the cardinal symptoms of this disorder. Through a review of previous studies that measured abnormal self-knowledge in individuals with clinical MDD, several abnormalities that distinguish patients with MDD as well as those at risk of the illness were revealed. We also pointed out several possible directions for future clinical studies based on previous findings. Overall, this review extends our understanding of the relationship between self-knowledge and depression.

## Author Contributions

YLo wrote the paper. YLe supervised the review and assisted in paper revision. YM assisted in paper revision. PL assisted in paper revision. HL assisted in paper writing and funding supports. All authors were involved in revising the manuscript critically for important intellectual content and approved the final version of the manuscript.

### Conflict of Interest Statement

The authors declare that the research was conducted in the absence of any commercial or financial relationships that could be construed as a potential conflict of interest.
