# Supplementary material for: Review of Abnormal Self-Knowledge in Major Depressive Disorder
Source: Front Psychiatry. 2019 Mar 28;10:130. doi: 10.3389/fpsyt.2019.00130 (PMC6447699; doi:10.3389/fpsyt.2019.00130)
Supplement: Supplementary file 1 [file Data_Sheet_1.docx]

Supplementary materials


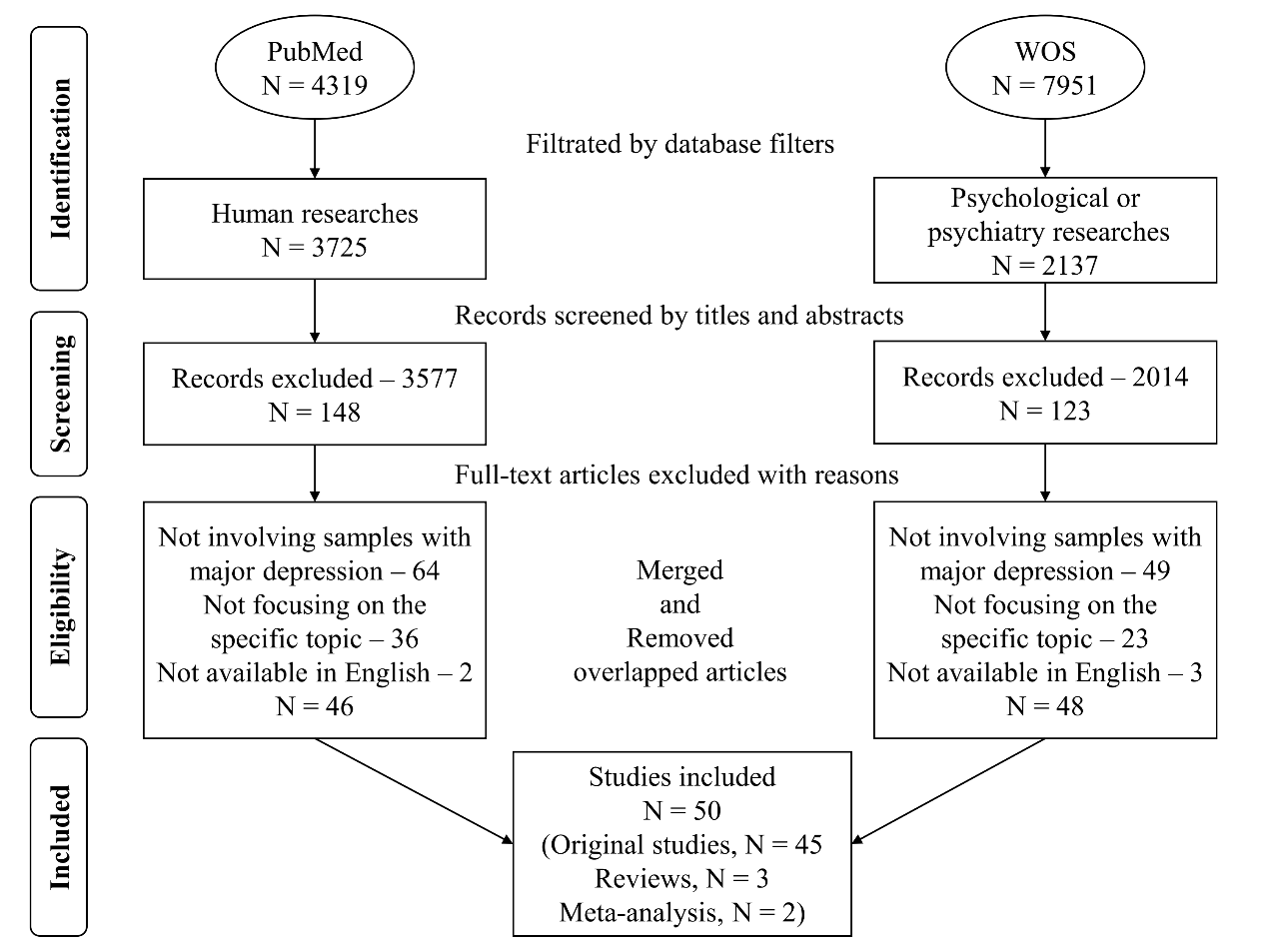


**Figure 1** Flow chat of procedure used for literature searching. Two databases, the PubMed and the Web of Science (WOS), were used.

**Table 1** Quality assessment of studies in Main Findings using the Newcastle-Ottawa Scale (NOS)

| Study | Description of population | Number of depressed individuals | Number of non-depressed controls | Selection | | | | Comparability | | Exposure/  Outcome | | |
| --- | --- | --- | --- | --- | --- | --- | --- | --- | --- | --- | --- | --- |
|  |  |  |  | 1 | 2 | 3 | 4 | a | b | 1 | 2 | 3 |
| Auerbach, R. P. et al. (2015) | Depressed adolescent with MDD | 22 | 28 | * | * | * | * | * | * | － | * | － |
| Bradley et al. (2016) | Adolescents with MDD | 20 | 15 | * | * | * | * | * | * | * | * | － |
| Dainer-best et al. (2017) | Adults with MDD | 21 | 23 | * | * | * | * | * | * | * | * | － |
| Dobson, K. S., et al. (1987) | Inpatients with depressed psychiatric | 24 | 29 | * | * | － | * | * | * | － | － | － |
| Frank et al. (2007) | Depressed patients with suicide ideation  Depressed patients without suicide ideation | 14  15 | 15 | * | * | － | * | * | － | * | * | － |
| Frank et al. (2008) | Currently depressed individuals  Formerly depressed individuals  Never depressed individuals | 29  35 | 38 | * | * | * | * | * | － | * | * | － |
| Gemar et al. (2001) | Current MDD  Formerly depressed patients | 32  23 | 27 | * | * | * | * | * | * | * | * | － |
| Grimm et al. (2009) | Subjects with an acute MDD episode | 25 | 25 | * | * | * | * | － | － | * | * | － |
| Jabben et al. (2014) | Outpatients with depressive symptom | 652 | 507 | * | * | * | * | * | * | * | * | － |
| J. E. Roberts et al. (2015) | Individuals with a past depressive episode | 28 | 33 | * | * | * | * | － | － | * | * | － |
| Jie et al. (2017) | Participants with high depression scores | 23 | 22 | * | * | * | * | * | * | * | * | － |
| Kesting et al. (2011) | Patients with depressive disorder | 21 | 59 | * | * | * | * | * | * |  | * | － |
| Kiang, M., et al. (2017) | Outpatients with nonpsychotic MDD | 16 | 16 | * | * | * | * | * | * | * | * | － |
| Lemmens et al. (2014) | Depressed patients | 87 | 30 | * | * | * | * | * | * | * | * | － |
| Lemogne, C., et al. (2009) | Patients with MDD | 15 | 15 | * | * | * | * | * | * | * | － | － |
| Li et al. (2017) | Depressive patients | 19 | 21 | * | * | * | * | * | － | * | * | － |
| Orchard, F., et al. (2018) | Depressed adolescent with MDD | 84 | 212 | * | * | * | * | * | － | * | － | － |
| Poulsen et al. (2009) | Depressed participants | 39 | 97 | * | * | * | * | * | * | * | * | － |
| Raedt et al. (2006) | Inpatients of MDD | 15 | 15 | * | * | * | * | * | － | － | － | － |
| Randenborgh et al. (2016) | Chronically depressed patients with an early onset  Chronically depressed patients with a late onset  Episodic depression | 17  13  29 | － | * | * | － | * | * | * | * | * | － |
| Risch et al. (2010) | First-onset currently depressed patients  Recurrently depressed patients  Remitted depressed patients | 24  28  33 | 34 | * | * | * | * | * | * | * | * | － |
| Romero et al. (2016) | Current MDD | 38 | 40 | * | * | * | * | * | * | * | * | － |
| Shestyuk et al. (2005) | MDD | 16 | 16 | * | * | * | * | * | * | * | * | － |
| Shestyuk et al. (2010) | Current MDD  Remitted MDD | 17  18 | 17 | * | * | * | * | * | * | * | * | － |
| Smeijers et al. (2017) | Remitted depressed patients | 75 | 75 | * | * | * | * | * | * | * | * | － |
| Thew, G. R. et al. (2017) | Current MDD | 26 | 26 | * | * | * | * | － | － | － | － | － |
| Van Tuijl et al. (2016) | Current MDD  Remitted MDD  Recovered MDD | 60  41  136 | 382 | * | * | * | * | * | * | * | * | － |
| Watson et al. (2008) | Dysphoric individuals | 17 | 10 | － | － | * | * | * | * | － | * | － |
| Yoshimura et al. (2010) | Clinician-diagnosed patients with unipolar major depression | 13 | 13 | * | * | * | * | * | * | * | * | － |

MDD: Major depressive disorder
